# Supplementary material for: Oxygen Tension Regulates Lysosomal Activation and Receptor Tyrosine Kinase Degradation
Source: Cancers (Basel). 2019 Oct 25;11(11):1653. doi: 10.3390/cancers11111653 (PMC6896048; doi:10.3390/cancers11111653)
Supplement: Supplementary file 1 [file cancers-11-01653-s001.pdf]

Supplementary Material: Oxygen Tension Regulates Lysosomal Activation and Receptor Tyrosine Kinase Degradation

Jaewoo Hong, Todd R. Wuest, Yongfen Min and P. Charles Lin

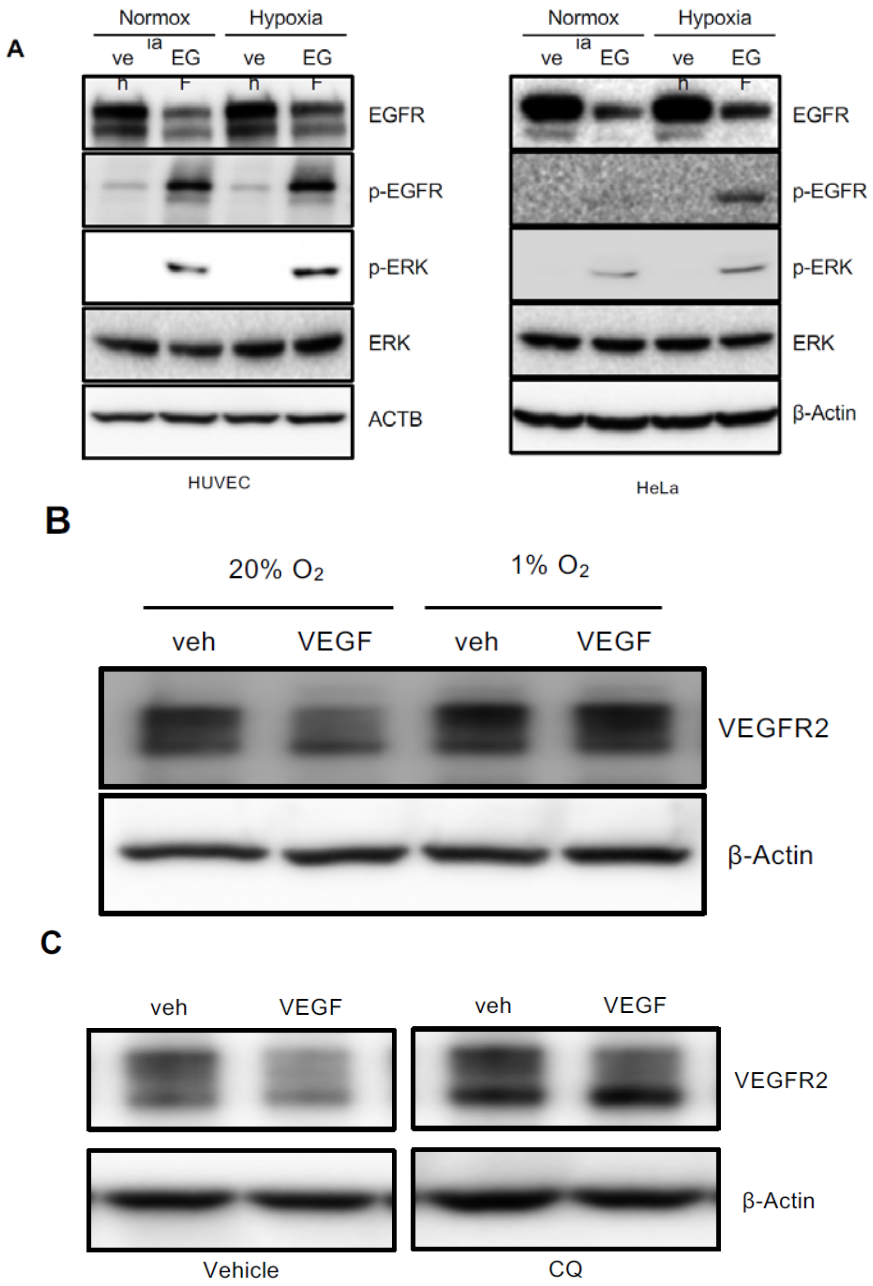

Figure S1. Serum-starved HUVECs and HeLa cells were cultured in either 20% or 1% oxygen in the presence or absence of 50 ng/mL of EGF for 5 h, followed by Western blot (**A**). Serum-starved HUVECs were cultured in either 20% or 1% oxygen in the presence or absence of 50 ng/mL of VEGF for 5 h, followed by Western blot (**B**). Serum-starved HUVECs were treated with 50 ng/mL of VEGF in the presence or absence of 5 µM of chloroquine (CQ) for 5 h (**C**).

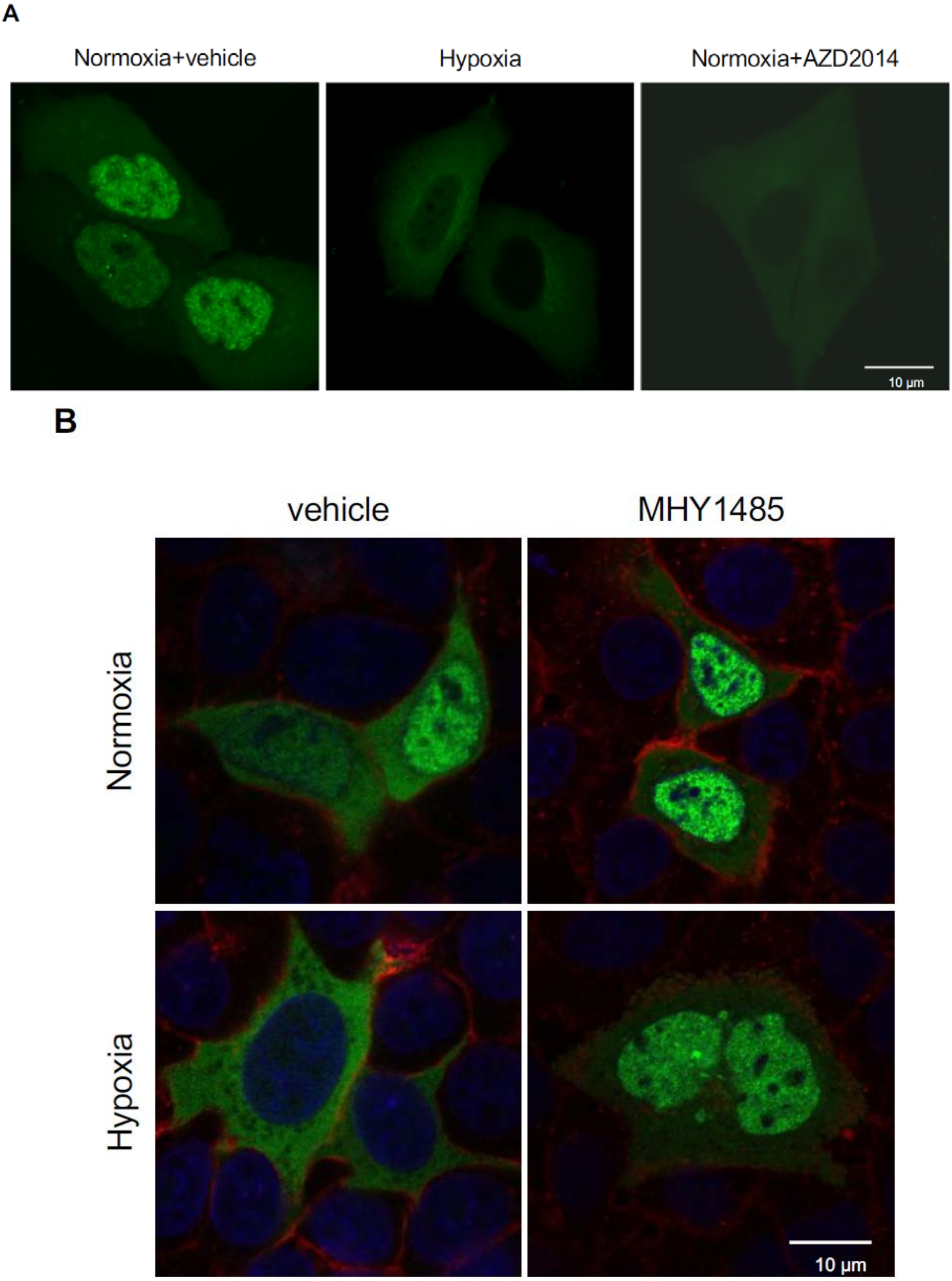

Figure S2. HeLa cells transfected with Myc tagged TFEB were incubated in 20% O_2 in the presence of absence of 10nM of AZD2014, or 1% O_2, for 5 h, followed by immunostaining for TFEB (**A**). HeLa cells transfected with Myc tagged TFEB were incubated in 20% or 1% O_2 in the presence of absence of 2 μM of MHY1485 for 4 h, followed by immunostaining for TFEB (green) and counter stained for wheat germ agglutinin (red) (**B**). Images were collected from confocal microscopy. Representative images were shown.

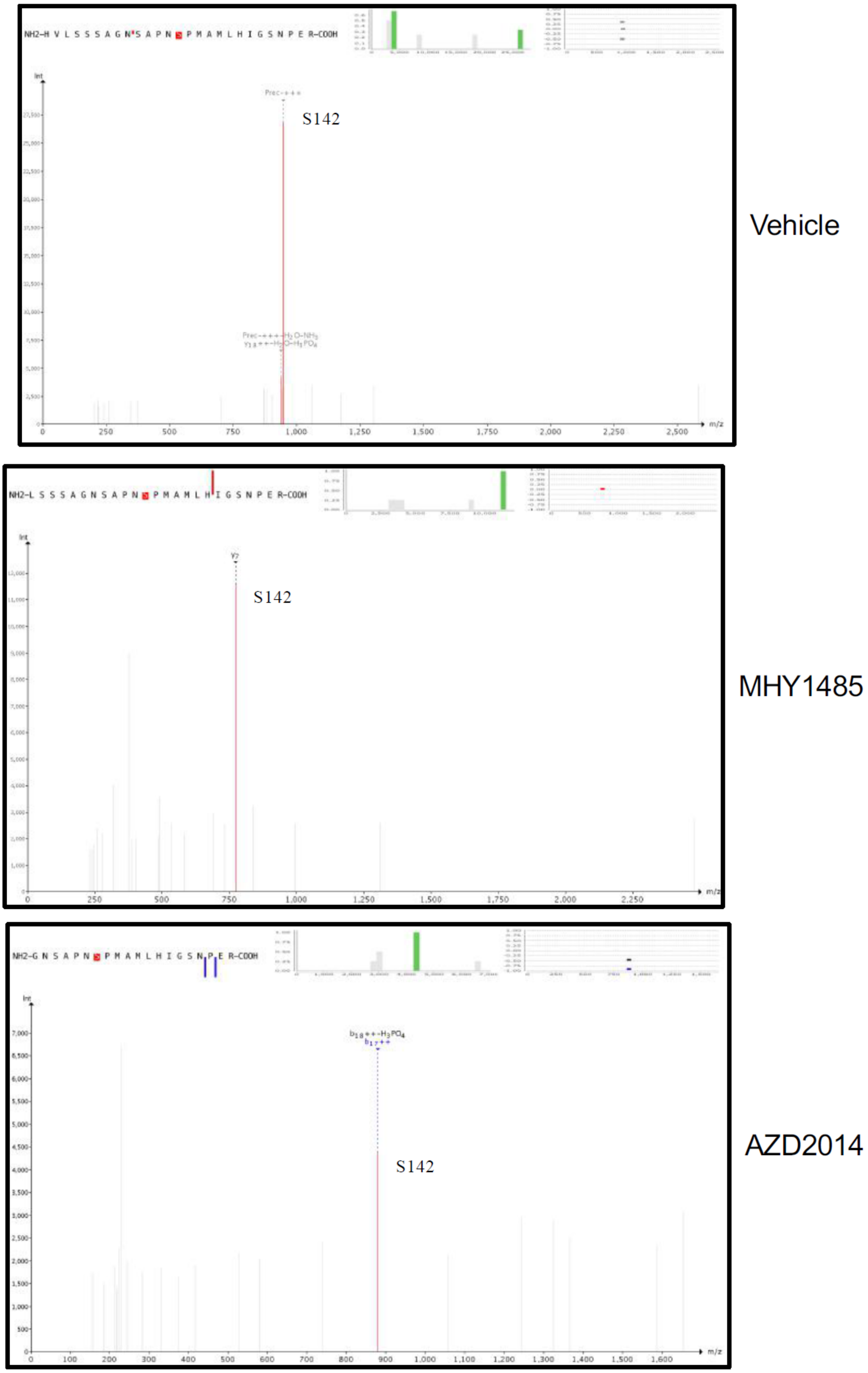

Figure S3. HeLa cells transfected with TFEB-myc expression vectors for 1 day, followed by stimulation with vehicle, 2 μM of MHY1485 or 10 nM of AZD2014 for 30 min. TFEB was immunoprecipitated with antibodies against the Myc tag, followed by mass-spectrometry analysis.

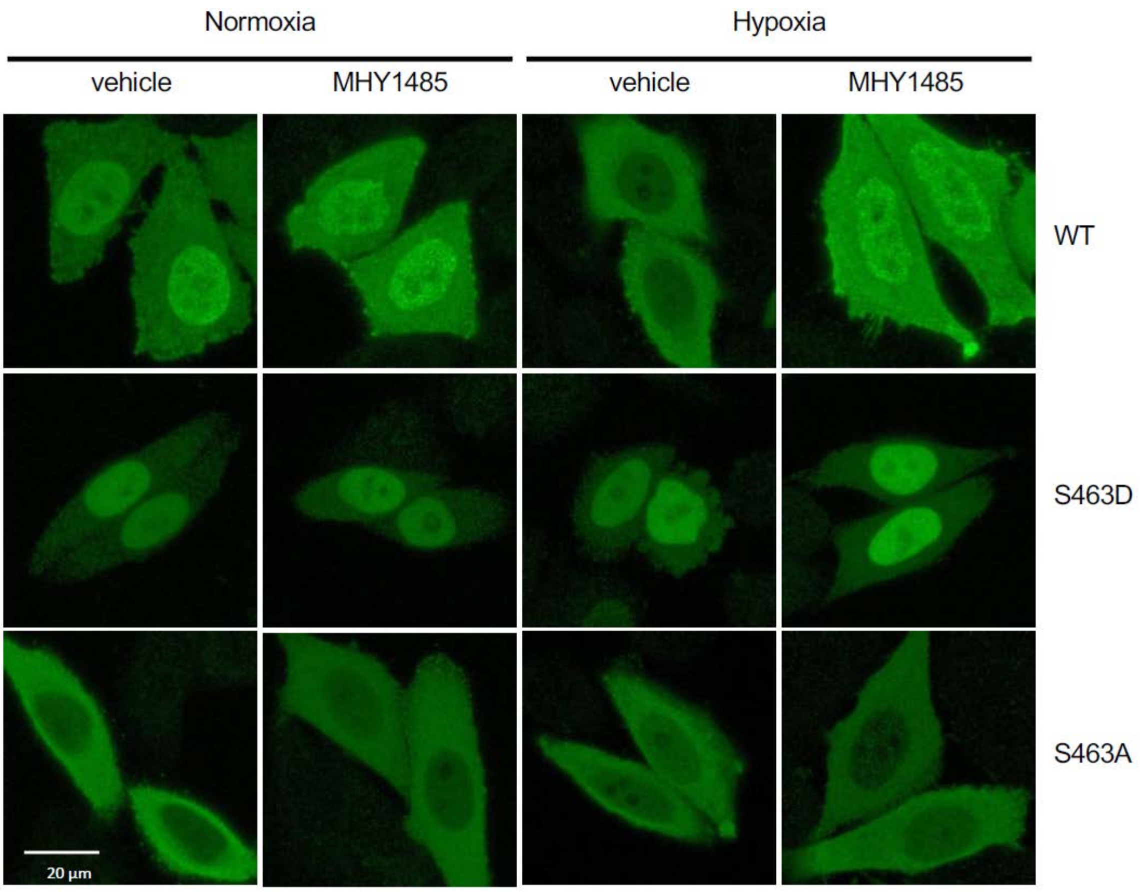

Figure S4. HeLa cells transfected with WT, S463A or S463D TFEB constructs were incubated in either 20% or 1% oxygen in the presence of absence of 2 μM of MHY1485 for 4 h, followed by immunostaining for TFEB. Images were collected from confocal microscopy. Representative images were shown.

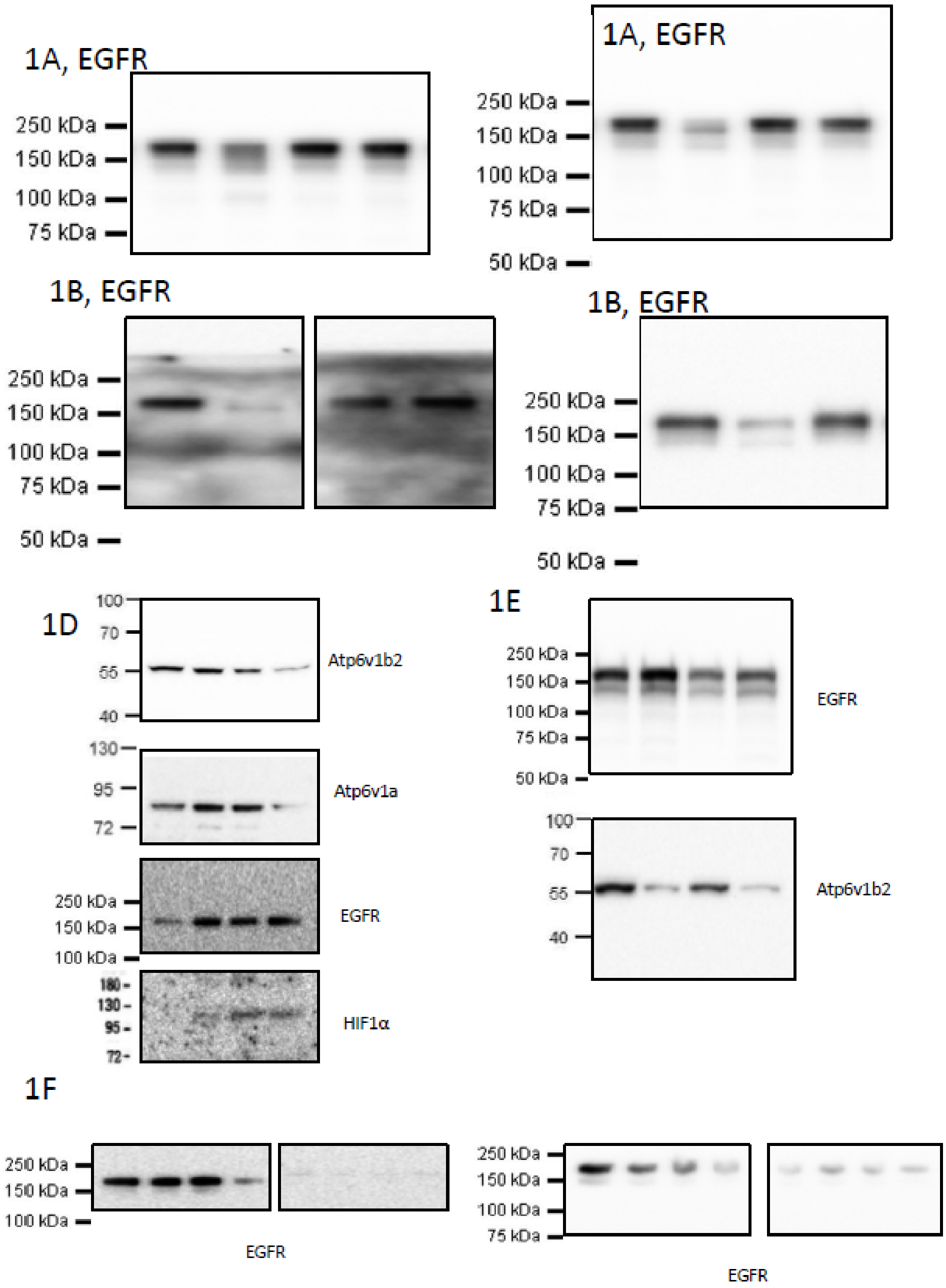

Figure S5. Raw data of Western blots from Figure 1.

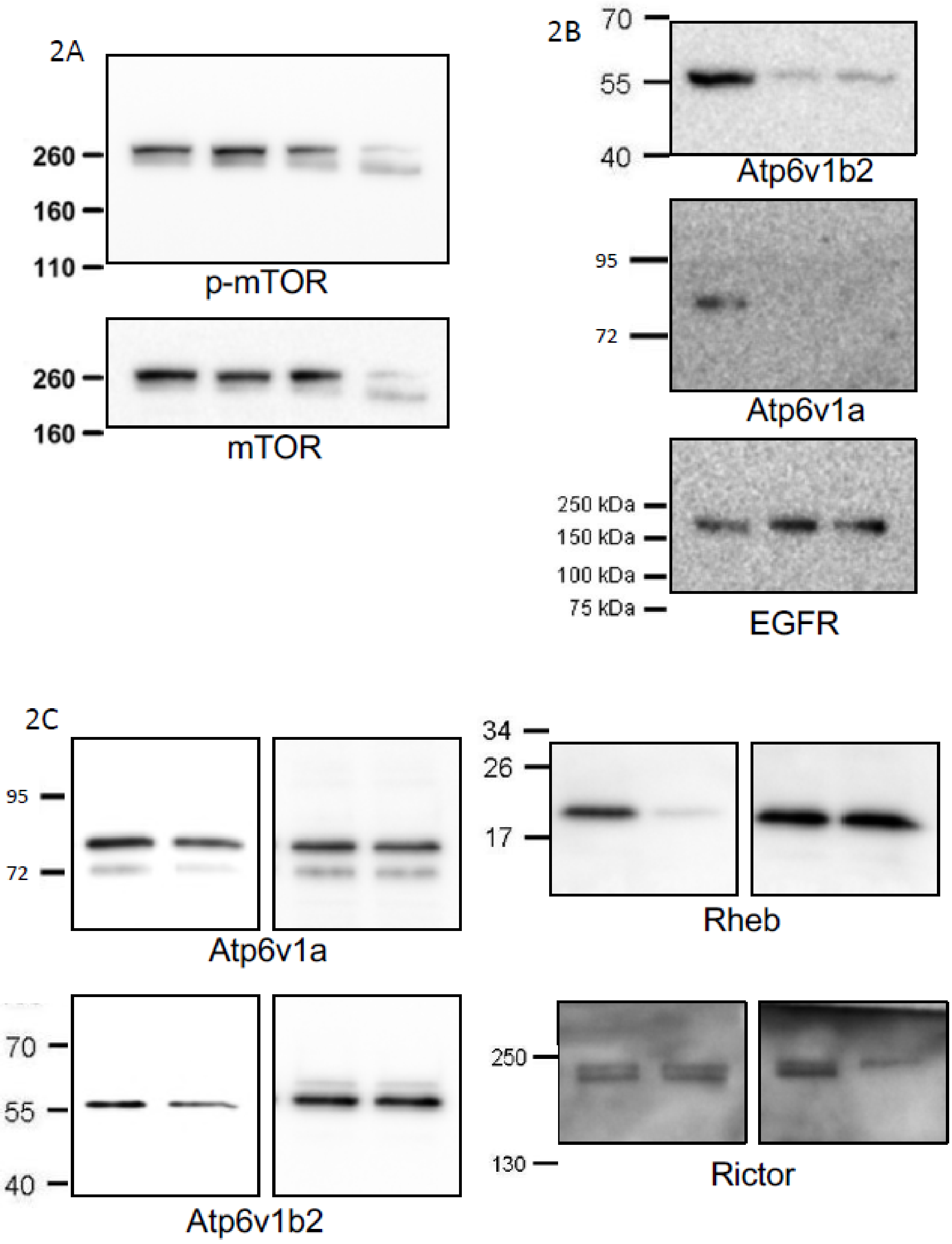

Figure S6. Raw data of Western blots from Figure 2.

© 2019 by the authors. Licensee MDPI, Basel, Switzerland. This article is an open access article distributed under the terms and conditions of the Creative Commons Attribution (CC BY) license (http://creativecommons.org/licenses/by/4.0/).
